# Chamber-Specific Structural, Fibrotic, and Molecular Remodeling of the Heart in Experimental Metabolic Syndrome

**DOI:** 10.3390/ijms27104427

**Published:** 2026-05-15

**Authors:** Óscar J. Arias-Mutis, Alexandra Bizy, Patricia Genovés, Johan E. Ortiz-Guzmán, Antonio Lucía-García, Amparo Ruiz-Saurí, César Ríos-Navarro, Luis Such-Miquel, Antonio Alberola, Francisco J. Chorro, Conrado J. Calvo, Manuel Zarzoso

**Affiliations:** 1 Department of Biomedical Sciences, CEU Cardinal Herrera University, 46115 Valencia, Spain; oscar.ariasmutis@uchceu.es (Ó.J.A.-M.); alexandra.bizy@uchceu.es (A.B.);; 2 Centro de Investigación Biomédica en Red de Enfermedades Cardiovasculares (CIBER-CV), 28029 Madrid, Spain; cesar.rios@uv.es (C.R.-N.); luis.such-miquel@uv.es (L.S.-M.); antonio.alberola@uv.es (A.A.); francisco.j.chorro@uv.es (F.J.C.); 3 Faculty of Health Sciences, University of Applied and Environmental Sciences (U.D.C.A.), Bogotá 111166, Colombia; johortiz@udca.edu.co; 4 Department of Physiology, Universitat de València, 46010 Valencia, Spain; 5 Department of Pathology, Universitat de València, 46010 Valencia, Spain; amparo.ruiz-sauri@uv.es; 6 Department of Physiotherapy, Universitat de València, 46010 Valencia, Spain; 7 Department of Medicine, Universitat de València, 46010 Valencia, Spain; 8 Instrumentation for Molecular Imaging Technologies Research Institute (I3M), Joint Research Center Spanish National Research Council–Polytechnic University of Valencia (CSIC-UPV), 46022 Valencia, Spain

**Keywords:** metabolic syndrome, cardiac structural remodeling, cardiac fibrosis, proteomics

## Abstract

Metabolic syndrome (MetS) drives cardiac remodeling and fibrosis, contributing to diastolic dysfunction and heart failure with preserved ejection fraction, but chamber-specific mechanisms remain poorly defined. New Zealand White rabbits were fed a high-fat/high-sucrose diet for 28 weeks to induce experimental MetS. Systemic phenotype, cardiac structure (echocardiography), myocardial fibrosis (Picrosirius red histology), myosin/collagen gene expression (qRT-PCR), and chamber-specific proteomics were assessed across left/right atria and ventricles. The model reproduced central obesity, glucose intolerance, dyslipidemia, and mild hypertension, with concentric left ventricular hypertrophy and selective ventricular fibrosis, as follows: increased collagen in left ventricle (LV) and right ventricle (RV), unchanged in atria. Ventricular *α*-myosin heavy-chain gene expression was upregulated, while collagen I and α-smooth muscle actin transcripts showed ventricular-specific downregulation. Proteomics revealed atrial metabolic and cytoskeletal adaptations with minimal extracellular matrix involvement; ventricles displayed early profibrotic cues (galectin-3 in LV), metabolic inefficiency (impaired glycolysis/ATP production in LV; lipid oxidation shift in RV), and diminished provisional matrix support. Conclusions: concentric LV hypertrophy and great vessel enlargement occurred without systolic/diastolic dysfunction; ventricular-selective fibrosis, α-myosin heavy-chain upregulation, type I collagen/α-smooth muscle actin downregulation, and chamber-specific proteomic changes showed atrial adaptation versus ventricular early profibrotic/metabolic inefficiency.

## 1. Introduction

Metabolic syndrome (MetS) has emerged as a major global health challenge, characterized by a cluster of interrelated metabolic disturbances including central obesity, dyslipidemia, hypertension and impaired glucose homeostasis [1]. Together, these abnormalities markedly increase the risk of type 2 diabetes and cardiovascular diseases [2,3]. At the mechanistic level, MetS reflects a chronic imbalance between energy intake and expenditure, leading to insulin resistance, compensatory hyperinsulinemia, activation of neurohormonal and prothrombotic pathways, and a persistent low-grade inflammatory state [4]. Beyond its systemic manifestations, MetS exerts direct deleterious actions on the heart, affecting both cardiomyocytes and the cardiac interstitium and thereby altering myocardial structure and function [5]. Clinically, individuals with MetS frequently display adverse left ventricular (LV) hypertrophy and subclinical systolic and diastolic dysfunction, even before overt heart failure develops [6,7].

A central hallmark of this “metabolic cardiomyopathy” is cardiac fibrosis, defined by excessive deposition and cross-linking of type I and III collagens within the myocardial extracellular matrix [8,9,10]. While these collagens are essential for preserving myocardial architecture and mechanical integrity, their abnormal accumulation increases ventricular stiffness, impairs relaxation, and progressively promotes diastolic dysfunction [11]. In the setting of MetS, fibrosis typically presents as diffuse interstitial and perivascular collagen expansion driven by chronic metabolic and hemodynamic stressors, including hyperglycemia, lipotoxicity, and insulin resistance [9]. Such diffuse fibrotic remodeling provides a structural substrate for the development of heart failure with preserved ejection fraction (HFpEF), a condition that disproportionately affects patients with MetS and obesity [12,13]. In both experimental and clinical studies, increased myocardial collagen content and cross-linking are tightly linked to LV stiffening, impaired filling, and adverse cardiovascular outcomes in cardiometabolic disease [13,14].

Importantly, fibrosis not only compromises ventricular mechanics but also disrupts the three-dimensional architecture of the myocardium, alters cell–cell and cell–matrix coupling, and creates conduction barriers that favor arrhythmogenesis [15]. In diet-induced obesity and MetS models, myocardial fibrosis and extracellular matrix expansion are associated with delayed repolarization, increased myocardial stiffness and electrical heterogeneity, all of which promote ventricular and atrial arrhythmias [13,16,17]. Nevertheless, despite accumulating evidence that MetS is linked to myocardial fibrosis, ventricular dysfunction and a higher burden of arrhythmias, the mechanistic pathways that connect metabolic derangements, structural remodeling, and proarrhythmic electrical changes remain incompletely defined. This is due, in part, to the complex, multifactorial nature of MetS and to the relative paucity of large animal models that simultaneously reproduce its metabolic, hemodynamic, fibrotic, and electrophysiological features [18,19,20,21,22].

In this context, we used a diet-induced MetS model in New Zealand White rabbits to perform an integrated in vivo and ex vivo characterization of cardiac remodeling. Specifically, we aimed to (i) characterize the systemic MetS phenotype (obesity, dyslipidemia, glucose intolerance, and blood pressure), (ii) quantify cardiac structural remodeling and fibrosis, and (iii) delineate chamber-specific gene expression and proteomic signatures in atrial and ventricular myocardium. This comprehensive approach is intended to provide insight into how MetS-associated fibrosis may contribute to arrhythmogenic substrate formation.

## 2. Results

### 2.1. Characterization of MetS

**Figure 1:**
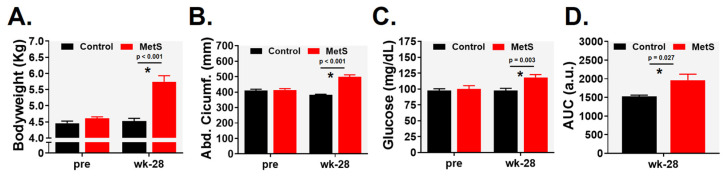
Morphological parameters and glucose regulation: (**A**) body weight, (**B**) abdominal circumference, (**C**) fasting glucose, and (**D**) area under the curve of the intravenous glucose tolerance test. Control (n = 7) and MetS (n = 8). Error bars: SEM. * *p* < 0.05.

Animals in the MetS group exhibited a progressive increase in body weight, reaching 27% higher values than controls by week 28 [5.74 (0.52) vs. 4.53 (0.22) kg; *p* < 0.001, r = 0.844; Figure 1A]. Concomitantly, abdominal circumference was significantly increased, confirming the development of central obesity [500 (32) vs. 383 (10) mm; *p* < 0.001, r = 0.932; Figure 1B]. At week 28, fasting blood glucose levels were also markedly elevated in the MetS group [118 (12) vs. 97 (8) mg·dL^−1^; *p* = 0.003, r = 0.708; Figure 1C]. This was accompanied by a significant increase in the area under the curve (AUC) during the glucose tolerance test [1962.50 (453.29) vs. 1529.29 (78.32) a.u.; *p* = 0.027, r = 0.567; Figure 1D], indicating impaired glucose tolerance.

**Figure 2:**
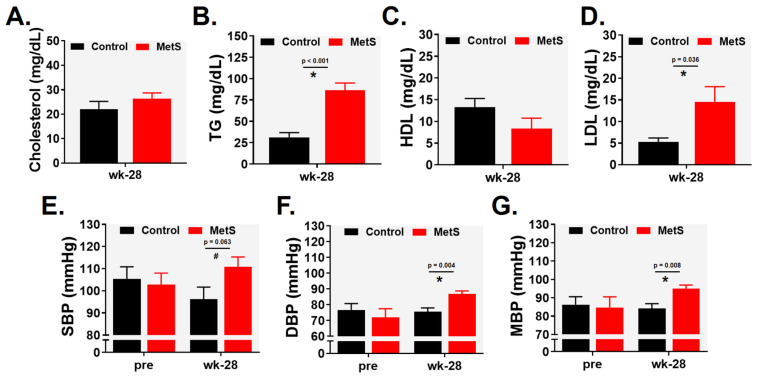
Lipid profile and blood pressure: (**A**) total cholesterol, (**B**) triglycerides, (**C**) high-density lipoprotein (HDL), (**D**) low-density lipoprotein (LDL), (**E**) systolic blood pressure (SBP), (**F**) diastolic blood pressure (DBP) and (**G**) mean blood pressure (MBP). Control (n = 7) and MetS (n = 8). Error bars: SEM. * *p* < 0.05, ^#^ *p* = 0.063.

The lipid profile of MetS rabbits showed characteristic features of metabolic syndrome. At week 28, these animals exhibited significantly elevated triglyceride levels [86.50 (23.31) vs. 31.28 (14.44) mg·dL^−1^; *p* < 0.001, r = 0.832; Figure 2B], a non-significant trend toward reduced HDL cholesterol (*p* = 0.075; Figure 2C), and markedly higher LDL cholesterol [14.50 (5.36) vs. 5.29 (2.43) mg·dL^−1^; *p* = 0.036, r = 0.544; Figure 2D]. Total cholesterol remained unchanged compared with controls (Figure 2A). In addition, diastolic blood pressure [86.8 (4.8) vs. 75.6 (5.2) mmHg; *p* = 0.004, r = 0.787; Figure 2F] and mean arterial pressure [95.0 (4.7) vs. 84.2 (5.8) mmHg; *p* = 0.008, r = 0.752; Figure 2G] were significantly increased in the MetS group, indicating the presence of mild hypertension at the end of the experimental protocol.

### 2.2. Cardiac Structural Remodeling

**Figure 3:**
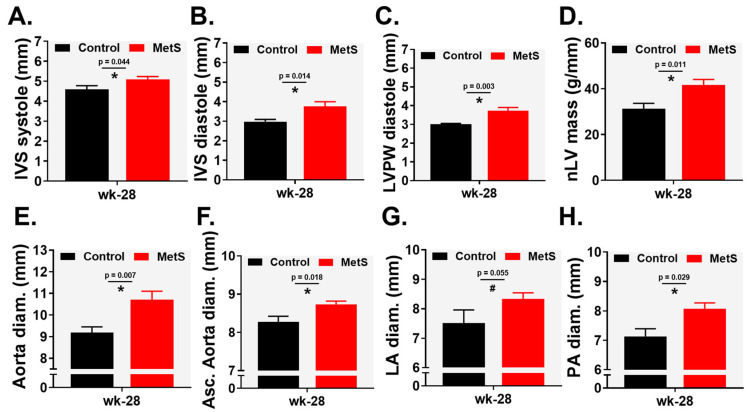
Echocardiographic study using standardized two-dimensional (2D), M-mode, and Doppler techniques: (**A**) interventricular septum (IVS) in systole, (**B**) IVS in diastole, (**C**) left ventricle posterior wall (LVPW) in diastole, (**D**) normalized LV mass, (**E**) aorta diameter, (**F**) ascending aorta diameter, (**G**) left atria (LA) diameter, and (**H**) pulmonary artery (PA) diameter. Control (n = 7) and MetS (n = 8). Error bars: SEM. * *p* < 0.05, ^#^ *p* = 0.063.

Echocardiographic evaluation at week 28 revealed structural cardiac remodeling in the MetS group. Interventricular septum (IVS) thickness was significantly increased during both systole [5.10 (0.38) vs. 4.58 (0.49) mm; *p* = 0.044, r = 0.526; Figure 3A] and diastole [3.76 (0.66) vs. 2.97 (0.31) mm; *p* = 0.014, r = 0.620; Figure 3B]. Left ventricular posterior wall (LVPW) thickness in diastole was also markedly elevated [3.72 (0.51) vs. 3.01 (0.11) mm; *p* = 0.003, r = 0.706; Figure 3C]. Consequently, normalized left ventricular mass (nLV mass) was significantly higher in MetS animals [41.58 (6.92) vs. 31.18 (6.51) g·mm^−1^; *p* = 0.011, r = 0.637; Figure 3D]. In addition, diameters of the aorta [10.72 (1.09) vs. 9.18 (0.68) mm; *p* = 0.007, r = 0.659; Figure 3E], ascending aorta [8.73 (0.20) vs. 8.27 (0.32) mm; *p* = 0.018, r = 0.623; Figure 3F], and pulmonary artery (PA) [8.06 (0.57) vs. 7.27 (0.68) mm; *p* = 0.029, r = 0.567; Figure 3H] were significantly enlarged in the MetS group. Left atrial (LA) diameter showed a non-significant trend toward enlargement (*p* = 0.055; Figure 3G). No difference was found at week 28 between control and MetS animals in hemodynamics, systolic, and diastolic function (Table S1).

### 2.3. Cardiac Fibrosis

**Figure 4:**
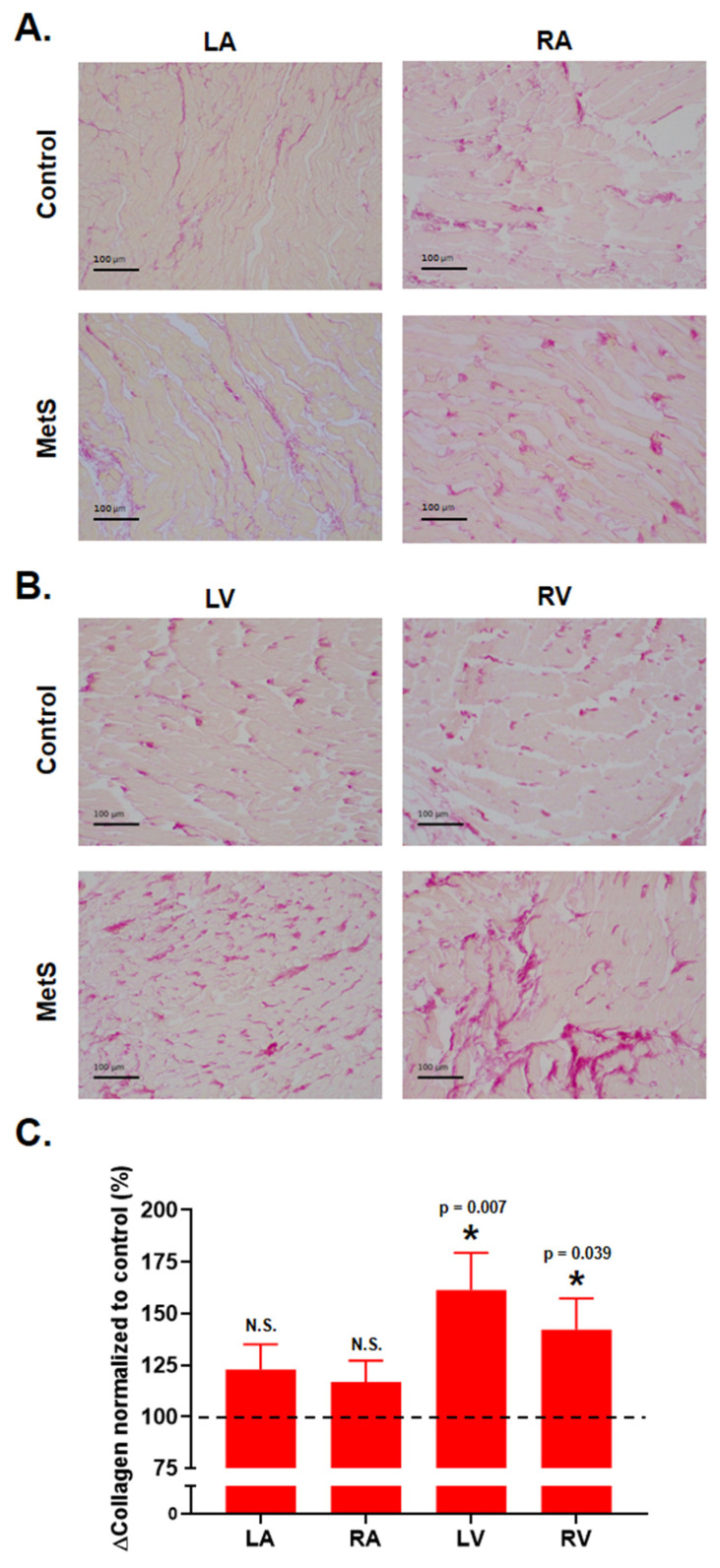
Increase in total collagen deposition across cardiac regions in Picrosirius red stained slices: (**A**) representative photographs of LV (**left**) and RV (**right**) in control (**top**) and MetS (**bottom**) under bright-field microscopy; (**B**) representative photographs of LV (**left**) and RV (**right**) in control (**top**) and MetS (**bottom**) under bright-field microscopy; (**B**) total collagen in left ventricle (LV); (**C**) increase in total collagen in right ventricle across all cardiac regions studied. Control (N = 5, n = 25) and MetS (N = 5, n = 25). N animals, n samples. Error bars: SEM. Dashed line represents controls (100%). * *p* < 0.05.

Histological examination revealed increased myocardial fibrosis in the MetS group at week 28. Total collagen content, quantified in Picrosirius red-stained sections, was significantly elevated in both the right ventricle (RV) [42.2 (75.3)%; *p* = 0.039, r = 0.293; Figure 4B,C] and the left ventricle (LV) [61.4 (90.3)%; *p* = 0.007, r = 0.379; Figure 4A,B]. In contrast, no significant difference in total collagen was observed in the atria (Figure 4A,C).

### 2.4. Gene Expression Across Cardiac Chambers

**Figure 5:**
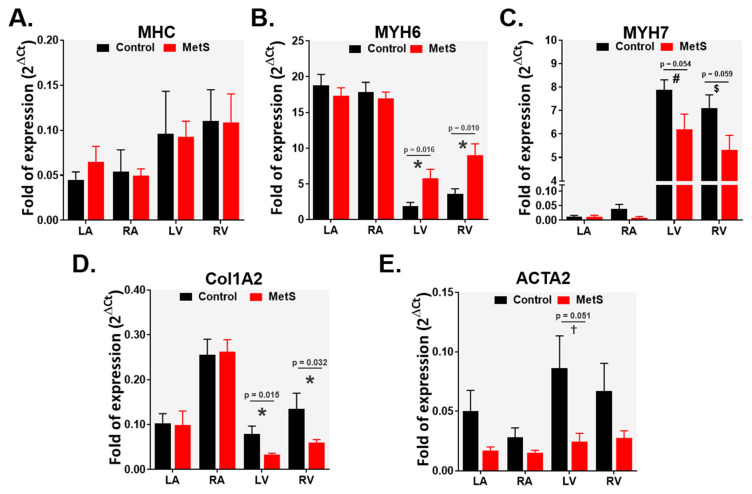
Gene expression of fibrosis and hypertrophy markers in all cardiac chambers: mRNA levels of (**A**) total myosin heavy-chain (MHC), (**B**) α-myosin heavy-chain isoform (MYH6), (**C**) β-myosin heavy-chain isoform (MYH7), (**D**) collagen type I alpha 2 chain (Col1A2), and (**E**) α-smooth muscle actin (ACTA2). Control (n = 6) and MetS (n = 6). Data normalized to GAPDH as the housekeeping gene. Error bars: SEM. * *p* < 0.05, ^#^ *p* = 0.054; ^$^ *p* = 0.059; ^†^ *p* = 0.051.

Gene expression analysis by quantitative real-time PCR revealed chamber-specific changes in myocardial gene expression in the MetS group at week 28. The α-myosin heavy-chain isoform (MYH6) was significantly upregulated in the left ventricle (LV; *p* = 0.016, r = 0.675) and right ventricle (RV; *p* = 0.010, r = 0.701) of MetS animals compared with controls (Figure 5B). In contrast, MYH6 expression in the atria (LA and RA) showed no differences between groups. The β-myosin heavy-chain isoform (MYH7) exhibited a non-significant trend toward lower expression in the LV (*p* = 0.054) and RV (*p* = 0.059; Figure 5C). As expected, MYH7 levels were substantially higher in the ventricular chambers than in the atria in both control and MetS groups, consistent with the known chamber-specific predominance of β-MHC in ventricular myocardium. Atrial MYH7 expression remained unaffected by MetS. Total myosin heavy-chain (MHC) expression did not differ significantly across chambers or between groups (Figure 5A). In parallel, collagen type I alpha 2 chain (Col1A2) mRNA levels were significantly lower in both the LV (*p* = 0.015, r = 0.627) and in the RV (*p* = 0.032, r = 0.551; Figure 5D) of MetS rabbits, whereas atrial Col1A2 expression was comparable between groups. Similarly, α-smooth muscle actin (ACTA2) expression displayed a strong trend toward downregulation in the LV (*p* = 0.051; Figure 5E), with numerically lower values also observed in the RV (though not reaching statistical significance). Notably, despite the observed downregulation of Col1A2 and the trend in ACTA2 transcripts, histological analysis revealed a significant increase in interstitial and perivascular collagen deposition in the ventricular myocardium. Atrial levels remained unchanged.

### 2.5. Proteomic Study in Cardiac Tissue

**Figure 6:**
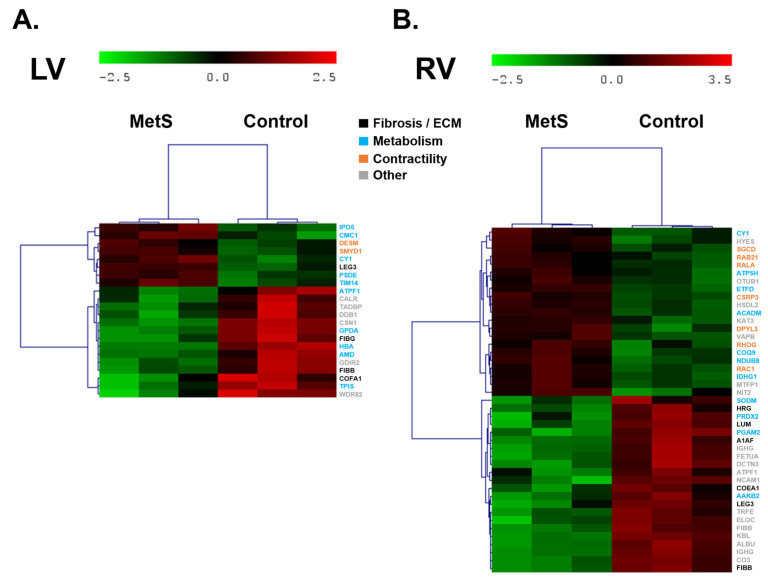
Heatmaps showing differential protein expression in ventricular tissue: (**A**) differentially expressed proteins identified in the left ventricle (LV) between control animals (C, right) and animals with metabolic syndrome (MetS, left); (**B**) differentially expressed proteins identified in the right ventricle (RV) between control animals (C, right) and animals with metabolic syndrome (MetS, left). Color code of the proteins correspond to the main process or function. Control (n = 3) and MetS (n = 3).

**Figure 7:**
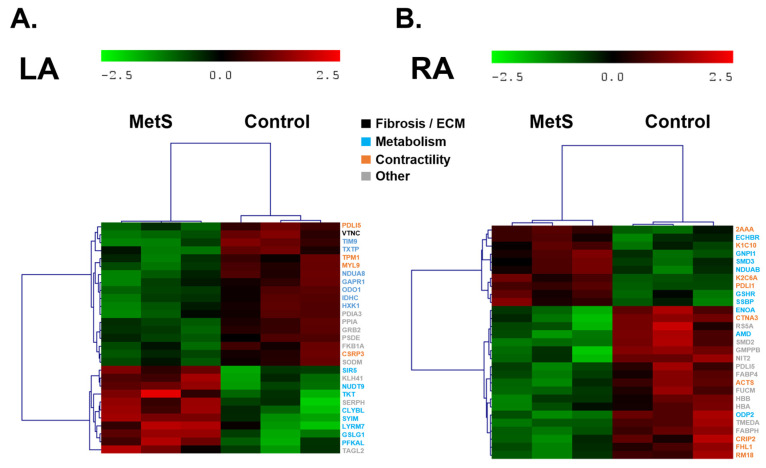
Heatmaps showing differential protein expression in atrial tissue: (**A**) differentially expressed proteins identified in the left atrium (LA) between control animals (C, right) and animals with metabolic syndrome (MetS, left); (**B**) differentially expressed proteins identified in the right atrium (RA) between control animals (C, right) and animals with metabolic syndrome (MetS, left). Color code of the proteins correspond to the main process or function. Control (n = 3) and MetS (n = 3).

Proteomic profiling of atrial and ventricular myocardium identified a total of 1297 distinct proteins across all cardiac chambers. Differential expression analysis relative to controls revealed chamber-specific alterations in the MetS group at week 28 (Figure 6 and Figure 7, Table S2). Protein names displayed in the text and heatmaps correspond to UniProt accessions from rabbit when directly identified; otherwise, orthologous proteins from closely related species were selected based on sequence homology and used for consistent nomenclature.

In the LV, 22 proteins were differentially expressed (Figure 6A), showing early profibrotic/inflammatory signaling combined with metabolic inefficiency, without evidence of advanced fibrosis or widespread extracellular matrix (ECM) remodeling. Upregulation of galectin-3 (LEG3) may indicate incipient proinflammatory/profibrotic cues promoting macrophage recruitment and myofibroblast activation, while downregulation of matrix proteins (FIBG, FIBB, and COFA1), suggested reduced early fibrin scaffold formation and fibrillar collagen deposition rather than active fibrotic buildup. In terms of metabolic processes, the LV showed preserved or enhanced components of the mitochondrial electron transport chain (CY1), and a downregulation of proteins involved in glycolysis and mitochondrial ATP synthesis (e.g., GPDA and ATPF1), which may suggest impaired glycolytic flux and reduced mitochondrial ATP-generating capacity under chronic metabolic stress. Proteins involved in contractility and muscular function showed targeted upregulation of desmin (DESM) and SMYD1, with no major contractile proteins downregulated, suggesting compensatory reinforcement of cytoskeletal and myofibrillar stability to counteract early metabolic and inflammatory stress. These findings suggest that the LV may be undergoing an initial maladaptive phase with incipient inflammatory/profibrotic cues and metabolic derangement, prior to evident structural fibrosis or advanced ECM remodeling.

In the right ventricle (RV), proteomic analysis identified differential expression of 43 proteins (Figure 6B), reflecting a compensatory metabolic shift toward fatty acid β-oxidation, accompanied by reduced ECM support and subtle inflammatory/matrix disorganization, without signs of advanced fibrosis or extensive ECM remodeling. No classical profibrotic proteins were upregulated; instead, marked downregulation of matrix-supporting proteins—including fibrinogen beta chain (FIBB), lumican (LUM), collagen alpha-1 (VI) chain (COEA1), histidine-rich glycoprotein (HRG), alpha-1-antifibrinogen (A1AF), and galectin-3 (LEG3)—suggested impaired provisional matrix formation, collagen fibrillogenesis, and overall matrix organization rather than active fibrotic deposition. Metabolically, the RV appeared to show upregulation of proteins supporting mitochondrial β-oxidation and respiratory chain function (ACADM and CY1), possibly indicating a compensatory increase in lipid catabolism capacity in response to metabolic stress. Downregulation of glycolytic and antioxidant enzymes (PGAM2 and SODM) further supported selective reprogramming favoring fatty acid oxidation over glycolysis. Proteins involved in contractility and muscular function showed targeted upregulation (CSRP3), suggesting reinforcement of actin dynamics and myofibril stability, while no major contractile proteins were downregulated in this set. These findings indicate that the RV may be undergoing a compensatory metabolic adaptation with early matrix disorganization and enhanced cytoskeletal/sarcolemmal integrity, prior to progressive structural remodeling in this model.

In the LA, 29 proteins were differentially expressed between controls and MetS (Figure 7A). The proteomic profile showed a distinct pattern predominantly linked to metabolic adaptation and cellular stress responses, with no prominent signature of fibrosis or ECM remodeling. Upregulated proteins were mainly associated with enhanced metabolic flux and mitochondrial function (PFKAL and TKT), protein synthesis and trafficking (SYIM), and partial cytoskeletal/muscle differentiation support (TAGL2, SERPH, and KLH41). In contrast, downregulated proteins encompassed vitronectin (VTNC) involved in ECM adhesion and provisional matrix support, mitochondrial protein import and assembly (TIM9), glycolytic and TCA cycle capacity (HXK1 and ODO1), chaperone-mediated protein folding and stress response (PDIA3 and SODM), receptor tyrosine kinase signaling (GRB2), and additional mitochondrial/Golgi functions. Proteins directly involved in contractility and muscular function showed consistent downregulation, including tropomyosin alpha-1 (TPM1), myosin regulatory light chain 9 (MYL9), cysteine- and glycine-rich protein 3 (CSRP3), and PDZ/LIM-domain protein 5 (PDLI5), indicating impaired actin filament stabilization, contractile regulation, and Z-disk scaffolding in cardiomyocytes. This pattern points to reduced cellular protective mechanisms, impaired mitochondrial homeostasis, and altered cytoskeletal integrity, with diminished contractile capacity and only limited adaptive cytoskeletal remodeling in the LA.

In the RA, 29 proteins showed differential expression (Figure 7B), primarily involving proteins related to cytoskeletal organization, mitochondrial respiratory chain function, and metabolic adaptation, with no prominent direct indicators of fibrosis or ECM remodeling. Upregulated proteins were predominantly linked to structural and cytoskeletal support (PDLI1, K1C10, and K2C6A) as well as mitochondrial electron transport components and redox balance (NDUAB and GSHR). In contrast, downregulated proteins encompassed several associated with mitochondrial metabolism and pyruvate dehydrogenase function (ODP2), glycolysis and enolase activity (ENOA), protein folding/chaperone activity (PDLI5), lipid transport (FABP4), ribosomal/mitochondrial assembly, hemoglobin subunits, and additional stress-related proteins. Proteins directly involved in contractility and muscular function showed consistent downregulation (ACTS, CTNA3, CRIP2, FHL1, and PDLI5), indicating reduced actin filament dynamics, Z-disk scaffolding, and contractile apparatus support in cardiomyocytes. In contrast, upregulation of intermediate filament keratins (K1C10 and K2C6A) and PDZ/LIM domain protein 1 (PDLI1) may suggest partial compensatory reinforcement of structural integrity. Overall, the proteomic signature in the right atrium highlights cytoskeletal reinforcement and mitochondrial respiratory chain maintenance alongside compromised metabolic efficiency and cellular protective mechanisms, rather than overt fibrotic or ECM remodeling, with diminished contractile capacity.

## 3. Discussion

The combination of progressive weight gain, increased abdominal circumference, impaired glucose tolerance, hypertriglyceridemia, elevated LDL, reduced HDL tendency, and mild hypertension indicates that the rabbits developed a robust MetS phenotype, consistent with criteria commonly used to validate diet induced MetS in rodents and rabbits [18,19,23,24,25]. High carbohydrate/high fat or Western-type diets similarly induce central obesity, dyslipidemia, hypertension, and glucose intolerance in rats and mice, and these systemic alterations are considered key drivers of cardiovascular and multiorgan remodeling [18,23,25,26,27]. In rabbits, high-fat/high-sucrose regimes closely comparable to ours reproduce abdominal obesity, elevated triglycerides and LDL, decreased HDL, and hypertension, supporting the translational value of this species for MetS research [19,28]. The modest elevation in diastolic and mean arterial pressure at week 28 is consistent with the early hypertensive phase reported in these rabbits and in Western-diet rodent models, reinforcing the translational value of this large-animal system [19,27,29]. Although these values remain within the broad physiological range reported for rabbits, the relative elevation induced by the high-fat/high-sucrose diet is biologically relevant in the context of metabolic syndrome. This modest hypertensive shift is consistent with previous diet-induced rabbit models of MetS, in which mild increases in blood pressure (systolic, diastolic, and mean arterial pressure) have been associated with endothelial dysfunction, insulin resistance, dyslipidemia, and early cardiac remodeling, even in the absence of severe hypertension [19,28,30]. The associated hepatic steatosis and metabolomic alterations described previously in this model further align with the multi organ involvement reported in other diet induced MetS models [18,19,23,25,28].

Echocardiographic findings of LV concentric remodeling—thickened interventricular septum and posterior wall, increased normalized LV mass, and dilatation of the aorta, ascending aorta, and pulmonary artery—agree with structural adaptations documented in high-fat diet rabbits and in HFHS rodents after comparable durations [23,27,31,32,33]. In HFHS rat and mouse models, early septal and posterior-wall thickening with increased diastolic wall thickness precedes systolic impairment, similar to the compensated phase observed here [27,29,31]. Great-vessel enlargement likely stems from pressure overload and/or endothelial dysfunction due to hyperlipidemia and hypertension, phenotypes also reported in sustained high-fat feeding in rabbits and other large-animal models 2022 [28,32,33,34]. The trend toward larger left atrial diameter is consistent with early structural adaptation to increased filling pressures and altered diastolic properties described in several long-term diet induced obesity or diabetic cardiomyopathy models [18,31,35,36].

Histological examination revealed a clear ventricular-predominant pattern of interstitial fibrosis in the MetS group at week 28. Total collagen content, quantified in Picrosirius red-stained sections, was significantly elevated in both the RV and LV, whereas atrial collagen deposition remained unchanged. This distribution aligns with that reported in diet-induced MetS and sustained high-fat or Western-diet models in rabbits, mice, and rats, where myocardial collagen accumulation—predominantly interstitial and perivascular—contributes to increased myocardial stiffness, cardiomyocyte hypertrophy, and diastolic dysfunction [23,27,33,35,36,37,38]. In these models, long-term high-sucrose/high-fat feeding is consistently associated with oxidative stress, inflammation, and dysregulation of extracellular-matrix (ECM) regulatory enzymes, including elevated lysyl oxidase activity that enhances collagen cross-linking [18,23,26,27,31]. Indeed, in a murine MetS model, increased lysyl oxidase-mediated cross-linking produced ventricular stiffness despite complex transcriptional alterations, underscoring that fibrotic remodeling in diet-induced MetS depends not only on collagen quantity but also on its post-translational modifications [26]. The selective ventricular involvement observed here, despite proteomic changes in the atria, indicates that the ventricles are particularly susceptible to the early fibrotic effects of metabolic overload. This chamber specificity is likely driven primarily by greater wall stress and pressure/volume loading rather than systemic metabolic factors alone; in rodent models, atrial fibrosis typically emerges later or requires additional stressors such as aging, neurohumoral activation, or sustained arrhythmic burden [18,27,29,31,35,36,37].

Gene-expression profiling by quantitative real-time PCR revealed chamber-specific transcriptional changes at week 28. MYH6 (α-MHC) was significantly upregulated in both ventricles (LV and RV), with a trend toward lower MYH7 (β-MHC) expression; atrial isoforms and total MHC levels remained unaltered. Ventricular type I collagen gene expression was downregulated (significant in LV, albeit trending in RV), and α-smooth muscle actin gene expression showed a strong downward trend in the LV, while atrial transcripts were unaffected. This ventricular-predominant shift toward a faster α-MHC phenotype contrasts with the canonical fetal MYH7-up/MYH6-down reprogramming in rodent models of pathological hypertrophy yet aligns with basal β-MHC predominance in rabbit and human ventricles and shows the heterogeneous responses in diet-induced MetS models across species [27,31,36,39,40]. MHC isoform switching in high-fat-diet models is time- and load-dependent, driven by metabolic substrate shifts, oxidative stress, and neurohormonal status [18,25,36]. The α-MHC increase likely represents a compensatory adaptation enhancing contractile velocity and energetic efficiency under early metabolic stress. Concomitantly, the paradox of increased histological collagen despite reduced type I collagen gene expression and trending-down ACTA2 transcripts mirrors diet-induced MetS models in mice and rats, where extracellular-matrix remodeling relies on altered turnover, cross-linking, and matrix metalloproteinase activity rather than transcriptional upregulation [18,23,26,27,41]. This supports post-transcriptional and post-translational mechanisms dominating fibrotic progression in chronic MetS. The lack of atrial transcriptional changes is consistent with modest atrial remodeling, indicating relative atrial sparing during early-to-mid-term metabolic overload.

In the diet-induced MetS model, proteomic analysis across the four cardiac chambers revealed chamber-specific remodeling patterns dominated by metabolic reprogramming and early inflammatory/profibrotic cues, with no evidence of advanced fibrosis or extensive ECM remodeling in any chamber. In both atria, the signatures were primarily metabolic and stress-response oriented, with no prominent fibrotic/ECM involvement; upregulated proteins supported enhanced glycolytic flux, pentose phosphate pathway activity, mitochondrial function, and respiratory chain assembly, while downregulation affected mitochondrial protein import, TCA cycle/glycolytic capacity, chaperone-mediated folding, antioxidant defense, cytoskeletal scaffolding, and lipid handling—indicating compensatory energy adaptation alongside reduced cellular protection and structural support. In contrast, ventricular chambers showed early profibrotic/inflammatory signals: the LV exhibited upregulation of galectin-3 alongside downregulation of provisional matrix proteins and collagen I, suggesting incipient proinflammatory/profibrotic milieu without mature collagen deposition. Interestingly, the proteomic profiling revealed a significant chamber-specific upregulation of galectin-3 in the left ventricle of MetS rabbits. Considering the well-established role of galectin-3 in promoting fibroblast activation, collagen deposition, and myocardial fibrosis [42], this finding provides molecular support for the increased interstitial and perivascular fibrosis observed in the histological analysis of the LV. Metabolically, LV preserved mitochondrial electron transport but showed impaired glycolysis and ATP production. The RV seemed to exhibit a compensatory profile characterized by enhanced capacity for fatty acid β-oxidation and preserved respiratory chain support, together with reduced levels of some glycolytic enzymes, redox balance proteins, and matrix-supporting components (fibrinogen, lumican, and collagen VI), possibly indicating selective lipid catabolism preference and reduced provisional ECM organization. Collectively, atria demonstrate mainly metabolic/cytoskeletal adaptations with minimal ECM engagement, whereas ventricles reveal early inflammatory/profibrotic cues (via galectin-3 in LV), metabolic inefficiency (impaired glycolysis/ATP production in LV; lipid oxidation shift in RV), and diminished provisional matrix support—highlighting asymmetric chamber vulnerability and compensatory mechanisms prior to progressive structural remodeling. In Western-diet–fed Wistar rats and HFHS mice, large-scale myocardial proteomics identified early enrichment of pathways governing energy metabolism (glycolysis support and fatty-acid β-oxidation), cytoskeletal organization, mitochondrial redox balance, and substrate utilization, often preceding overt systolic dysfunction or macroscopic hypertrophy [27,31,35,39,43,44]. Ventricular predominance of mitochondrial and metabolic adaptations aligns with “metabolic cardiomyopathy” in diet-induced MetS, where shifts in substrate preference, β-oxidation capacity, and oxidative-stress defense constitute central compensatory mechanisms [18,23,35]. Downregulation of sarcomeric proteins and chaperones further suggests incipient contractile vulnerability and impaired proteostasis, as reported in long-term high-fat-diet rodent hearts [31,43]. The greater proteomic perturbation in the RV (43 vs. 22 proteins in the LV) likely reflects its thinner wall and heightened sensitivity to pressure/volume overload and fatty-acid-driven energetic stress, consistent with prior RV electrophysiological remodeling in this rabbit model [22]. In contrast, the more modest atrial changes correlate with absent fibrosis and limited transcriptional remodeling, indicating subtler or delayed adaptations in atrial myocardium at this early-to-mid-term stage of metabolic overload. To our knowledge, this constitutes the first comprehensive atrial-versus-ventricular proteome analysis in a medium to large animal MetS model.

Although causal links between fibrosis and arrhythmias were not directly tested, our findings implicate ventricular fibrosis as a key pro-arrhythmic substrate. Excessive extracellular matrix disrupts myocyte alignment and gap junction distribution, generating conduction slowing and spatial dispersion of refractoriness that favor re-entry—mechanisms tightly associated with increased ventricular arrhythmia risk in high-fat/high-sucrose models [22,37,38]. In the same NZW rabbit MetS model, LV hypertrophy coexists with marked right-to-left heterogeneity in action potential duration and dominant frequency during ventricular fibrillation, abolishing physiological activation gradients; this is at least partly driven by regional upregulation of repolarizing K^+^ channels in the RV and is sufficient to increase vulnerability to sustained ventricular arrhythmias even in the absence of overt systolic failure [22]. Similarly, in fructose- or Western-diet rodent models, myocardial fibrosis and inflammatory remodeling are accompanied by delayed repolarization, conduction abnormalities, and increased susceptibility to both atrial fibrillation and ventricular tachyarrhythmias [27,29,38,45]. The ventricular-restricted fibrosis observed here, combined with cytoskeletal and mitochondrial proteomic shifts that may affect Ca^2+^ handling, redox balance, and action potential properties, is therefore consistent with an enhanced arrhythmogenic substrate, providing a mechanistic bridge between structural remodeling and the heightened arrhythmogenesis reported in obese and diabetic hearts across species [15,22,31,37,46].

Several limitations should be considered when interpreting the present findings. First, while echocardiography provided standard structural and functional indices, more advanced diastolic parameters (e.g., strain imaging) or invasive hemodynamic assessments were not included and could have revealed subclinical changes in myocardial relaxation or filling pressures. Second, all structural, histological, gene expression, and proteomic analyses were conducted at a single time point (week 28), precluding evaluation of the temporal progression of cardiac remodeling, the onset and evolution of fibrosis, or potential reversibility at earlier or later stages of metabolic overload. Third, the proteomic analysis was conducted with a relatively small sample size per group, which may have limited statistical power to detect subtler chamber-specific differences or contributed to type II errors for some non-significant trends. Furthermore, the proteomic and gene expression analyses were performed on bulk myocardial tissue, lacking cell type–specific resolution (e.g., cardiomyocyte vs. fibroblast contributions). Finally, although the high-fat/high-sucrose rabbit model faithfully recapitulates several features of human MetS, species differences in cardiac physiology, fibrosis susceptibility, lipid handling, and inflammatory responses may restrict direct translation to clinical settings.

In summary, to our knowledge, this study is the first to combine structural, fibrotic, transcriptomic, and chamber-resolved proteomic analyses of cardiac remodeling in a diet-induced NZW rabbit MetS model. By revealing ventricular-predominant fibrosis, an atypical myosin isoform shift, and distinct atrial–ventricular proteomic adaptations—extending beyond the more uniform left ventricular changes typically reported in HFHS mice, rats, and minipigs—we establish a translational platform bridging rodent, porcine, and human MetS cardiomyopathy. These insights might help advance mechanistic understanding and highlight chamber-specific targets to mitigate fibrosis and arrhythmogenesis in metabolic syndrome.

## 4. Materials and Methods

### 4.1. Animals and Diet

Adult male New Zealand White rabbits (n = 18), aged 16–18 weeks and with an initial body weight of 4.55 (0.18) kg, were included in the study following a three-week acclimatization period under strictly controlled environmental conditions (ambient temperature 20 (1.5) °C, relative humidity 50 (5%), and a 12 h light/dark cycle), with ad libitum access to water and a daily ration of 120 g of standard maintenance chow (V2333-000, Ssniff, Soest, Germany). Upon completion of acclimatization, the animals were randomly allocated to two experimental groups: the control group (n = 9), which continued to receive the standard chow exclusively throughout the 28-week protocol, and the metabolic syndrome group (n = 9), which was fed ad libitum for 28 weeks with a high-fat, high-sucrose diet consisting of standard chow supplemented with 10% hydrogenated coconut oil and 5% lard (S9052-E020, Ssniff), and provided exclusively with a 15% aqueous sucrose solution as the drinking fluid [19].

### 4.2. Morphological, Plasma and Blood Pressure Measurements

Body weight, abdominal circumference, and body mass index (BMI) were recorded on a weekly basis throughout the experimental period. Fasting blood glucose levels and intravenous glucose tolerance tests (IVGTT) were conducted before diet administration and at week 28, following established protocols described previously [19]. Blood pressure was measured invasively via catheterization of the central auricular artery in conscious animals. Briefly, rabbits were gently restrained in a plastic holder, and topical local anesthesia (EMLA cream, AstraZeneca, Madrid, Spain) was applied to the cannulation site. The central auricular artery was then cannulated using an 18G Introcan catheter (B. Braun, Melsungen, Germany). Following cannulation, restraints were relaxed, allowing the animal to acclimatize quietly for 20–30 min. Arterial blood pressure was recorded directly through the catheter connected to a pressure transducer (Model 60-3003, Harvard Apparatus, Holliston, MA, USA) positioned at the level of the heart. The transducer signal was amplified and digitized using a PowerLab 2/26 data acquisition system (AD Instruments, Oxford, UK), with data acquired at a sampling rate of 1 kHz and registered in LabChart software (version 6, AD Instruments, Oxford, UK). Custom-developed software was employed for offline analysis, processing the final 5 min of a 20 min stable recording to determine systolic blood pressure, diastolic blood pressure, and mean arterial pressure (MAP). These measurements were performed before diet administration and at week 28. Blood samples were obtained at week 28 after a 7 h fasting period and analyzed for triglycerides, total cholesterol, high-density lipoprotein (HDL) and low-density lipoprotein (LDL) using standard enzymatic assays (Immunovet, Barcelona, Spain).

### 4.3. Echocardiographic Study

Animals were anaesthetized with propofol (10 mg kg^−1^) for induction, followed by maintenance with 2% isoflurane. After a 10 min stabilization period under anesthesia, echocardiographic evaluations were conducted at week 28. Transthoracic echocardiography was then performed following established protocols from prior studies [22], utilizing a Vivid S5 ultrasound system (GE Healthcare, Madrid, Spain) equipped with a 10S-RS phased-array transducer (4.5–11.5 MHz). Imaging modalities included two-dimensional (2D), M-mode, and Doppler techniques. Standard parameters related to hemodynamics, systolic function, diastolic function, and cardiac morphology were quantified as previously reported [33]. To mitigate anesthetic-induced hypothermia, body temperature was maintained using a thermal blanket heating device. During the echocardiographic examination, animals were positioned in lateral recumbency.

### 4.4. Histological Analysis

At the conclusion of the experimental period, rabbits were premedicated with ketamine (35 mg kg^−1^) and heparin (2500 IU), followed by euthanasia via intravenous overdose of sodium pentobarbital (100 mg kg^−1^). Hearts were then rapidly excised, rinsed in Tyrode solution, fixed in 4% formaldehyde, embedded in paraffin, sectioned at 5 μm thickness, and mounted onto double gelatin-coated glass slides. Tissue sections were stained with Picrosirius Red to quantify total collagen content. Specifically, sections were incubated in 0.1% Direct Red 80 (Sigma-Aldrich, St. Louis, MO, USA) dissolved in saturated picric acid solution to assess collagen deposition. Histological evaluation was performed in the myocardial region using a DMD108 microscope (Leica Microsystems, Wetzlar, Germany) with a 20× objective under bright-field microscopy. For each animal, five microphotographs per region of interest were acquired under standardized imaging conditions. Morphometric analysis was carried out using Image-Pro Plus 7.0 (Media Cybernetics, Rockville, MD, USA). Collagen deposition was quantified as the percentage of Picrosirius-Red-positive area relative to the total myocardial tissue area, allowing the assessment of interstitial fibrosis.

### 4.5. Gene Expression Analysis (qRT-PCR)

Tissue samples (approximately 2 mm^2^) from the right and left atria and ventricles were carefully dissected and separated immediately after heart excision, snap-frozen in liquid nitrogen, and stored at −80 °C until subsequent histological, gene expression, and proteomic analyses were performed. Total RNA was extracted from each cardiac chamber using TRIzol^®^ Reagent (Invitrogen, Carlsbad, CA, USA), and RNA purity and concentration were assessed spectrophotometrically. Complementary DNA (cDNA) was synthesized from the extracted RNA using the SuperScript III First-Strand Synthesis System (Thermo Fisher Scientific, Waltham, MA, USA). Relative gene expression levels of Col1a2, Acta2, Myh6, and Myh7 were quantified by real-time quantitative polymerase chain reaction (qRT-PCR) on an Applied Biosystems 7500 Real-Time PCR System, employing the 2ΔCt method with GAPDH as the endogenous reference (housekeeping) gene [22].

### 4.6. Proteomic Analysis

Proteomic analysis was performed to identify differentially expressed proteins in atrial and ventricular myocardium between the control and metabolic syndrome (MetS) groups using Swath technique. For sample preparation, approximately 50 mg of cardiac tissue from each chamber was homogenized in lysis buffer (7 M urea, 2 M thiourea, 4% CHAPS, 30 mM Tris-HCl, pH 8.5) using 2D Grinding Kit (GE Healthcare, Madrid, Spain). An aliquot of each sample (30 μg) was loaded into 1D_SDS_PAGE to evaluate protein profile from each condition. Gel fraction was cut and the sample was digested with sequencing grade trypsin (Promega, Madrid, Spain) as described elsewhere [47]. 750 ng of trypsin in 150 μL of ABC solution was used. The digestion was stopped with TFA (1% final concentration), a double extraction with ACN was performed, and all the peptide solutions and dried in a rotatory evaporator. Sample was re suspended with 25 μL of 2% ACN; 0.1% TFA. For the Swath LC-MS/MS analysis, 5 μL of each sample were loaded onto a trap column (NanoLC Column, 3 μ C18-CL, 75 μm × 15 cm; Eksigent, Barcelona, Spain) and desalted with 0.1% TFA at 3 μL·min^−1^ during 5 min. The peptides were loaded onto an analytical column (LC Column, 3 μ C18-CL, 75 μm × 12 cm, Nikkyo, Madrid, Spain) equilibrated in 5% acetonitrile 0.1% formic acid (FA). Peptide elution was carried out with a linear gradient of 5 to 35% B in 180 min (A: 0.1% FA; B: ACN, 0.1% FA) for at a flow rate of 300 nl·min^−1^. Peptides were analyzed in a mass spectrometer nanoESI qQTOF (5600 TripleTOF, ABSCIEX, Marlborough, MA, USA). The TripleTOF was operated in swath mode, in which a 0.050-s TOF MS scan from 350–1250 *m*/*z* was performed, followed by 0.080-s product ion scans from 350–1250 *m*/*z* on the 32 defined windows (3.05 s·cycle^−1^). The used Swath windows used were: 15 Da window widths from 450 to 1000 Da, 37 windows. Data analysis was performed using PeakView 2.1 software (SCIEX, Marlborough, MA, USA) for peak extraction and protein quantification from the SWATH-MS data. The resulting quantitative data were further processed and statistically analyzed with MarkerView 1.3 software (SCIEX). Protein identification was carried out against the UniProt Mammalia database (containing 1,905,360 protein entries). The comparisons of interest were control vs. MetS in each of the four cardiac chambers and analyses were conducted for each comparison with PCA, Welch *t*-test (for unequal variances), and HCT (hierarchical). All UniProt accessions are provided for precise identification and cross-referencing with public databases (Table S2).

### 4.7. Statistical Analysis

Data are presented as the mean (standard deviation, SD) unless otherwise stated. Normality of distribution was assessed with the Shapiro–Wilk test. For normally distributed data, between-group comparisons were performed using the unpaired Student’s *t*-test (two independent groups) or mixed-model ANOVA, as appropriate. In the proteomic analyses, Welch’s *t*-test (accounting for unequal variances) was applied, and the resulting *p*-values were adjusted for multiple comparisons using the Benjamini–Hochberg false discovery rate (FDR) procedure. Differences were considered statistically significant at *p* < 0.05.

## 5. Conclusions

In the present study, we characterized chamber-specific cardiac remodeling in New Zealand White rabbits fed a high-fat, high-sucrose diet for 28 weeks to induce experimental metabolic syndrome. By integrating echocardiography, histology, quantitative real-time PCR, and quantitative proteomics, we assessed structural, fibrotic, transcriptional, and proteomic alterations across the left atrium, right atrium, left ventricle, and right ventricle. Our main conclusions are as follows: (1) as previously reported, the diet-induced model faithfully reproduced key features of human MetS, including central obesity, impaired glucose tolerance, dyslipidemia, and mild hypertension; (2) concentric LV hypertrophy developed along with enlargement of the great vessels, without overt systolic or diastolic dysfunction at this stage; (3) myocardial fibrosis increased selectively in the ventricular myocardium, with no significant change in the atria; (4) ventricular-specific upregulation of α-myosin heavy-chain (MYH6) occurred alongside downregulation of collagen type I and α-smooth muscle actin transcripts; and (5) proteomic profiling uncovered chamber-distinct changes: while atria showed adaptive metabolic/cytoskeletal responses with minimal ECM involvement, ventricles displayed early profibrotic/inflammatory cues (via galectin-3 in LV), metabolic inefficiency, and reduced provisional matrix support at this early- to mid-term stage of metabolic overload.

## Data Availability

The original contributions presented in this study are included in the article/Supplementary Material. Further inquiries can be directed to the corresponding authors.

## Supplementary Materials

The following supporting information can be downloaded at: https://www.mdpi.com/article/10.3390/ijms27104427/s1.

## Abbreviations

The following abbreviations are used in this manuscript:

MetS	metabolic syndrome
NZW	New Zealand White
HFHS	high fat and high sucrose
LV	left ventricle
RV	right ventricle
LA	left atria
RA	right atria
HFpEF	heart failure with preserved ejection fraction
HDL	high-density lipoprotein
LDL	low-density lipoprotein
SBP	systolic blood pressure
DBP	diastolic blood pressure
MBP	mean blood pressure
IVS	interventricular septum
LVPW	left ventricle posterior wall
nLV	normalized left ventricle
MHC	myosin heavy chain
MYH6	α-myosin heavy-chain isoform
MYH7	β-myosin heavy-chain isoform
Col1A2	collagen type I alpha 2 chain
ACTA2	α-actin
ECM	extracellular matrix
BMI	body mass index
IVGTT	intravenous glucose tolerance test
SWATH	sequential window acquisition of all theoretical fragment ion spectra
LC-MS/MS	liquid chromatography tandem mass spectrometry
FA	formic acid
SEM	standard error of the mean
SD	standard deviation
PCR	polymerase chain reaction
ANOVA	analysis of variance
FDR	false discovery rate
