# Supplementary material for: Chamber-Specific Structural, Fibrotic, and Molecular Remodeling of the Heart in Experimental Metabolic Syndrome
Source: Int J Mol Sci. 2026 May 15;27(10):4427. doi: 10.3390/ijms27104427 (PMC13206778; doi:10.3390/ijms27104427)
Supplement: Supplementary file 1 [file ijms-27-04427-s001.zip › ijms-4248464-supplementary/2026-0424_Table S1.Echocardiographic characterization.pdf]

	Week 28	
	**Control**	**MetS**
**Haemodynamics**		
Heart rate (bpm)	252 (33)	231 (18)
Stroke volume (mL)	3.9 (0.9)	3.5 (1.2)
Cardiac output (mL·min^{-1})	962 (163)	827 (289)
**Systolic function**		
LV ejection fraction (%)	83 (2)	80 (5)
LV volume diastole (mL)	4.7 (1.1)	4.6 (1.4)
LV volume systole (mL)	0.9 (0.1)	0.8 (0.6)
LV fractional shortening (%)	48 (2)	46 (6)
LVID diastole (mm)	13.3 (1.1)	13.3 (1.7)
LVID systole (mm)	7.0 (0.5)	7.2 (1.3)
**Diastolic function**		
E wave vel. (cm·s^{-1})	0.57 (0.14)	0.60 (0.16)
A wave vel. (cm·s^{-1})	0.31 (0.14)	0.69 (0.15)
Isovolumic relax. time (ms)	37 (8)	42 (8)

Table S1. Echocardiographic characterization. Echocardiographic data at week 28 after high-fat, high-sucrose diet administration. LVID=left ventricle internal diameter, Control n=7, MetS n=8.
